# Synergistic effects of organic amendments and dual microbial inoculation on morphophysiological traits, nutrient uptake, and microbial symbiosis of black soybean (*Glycine max) in Inceptisol*

**DOI:** 10.1186/s12870-026-09244-9

**Published:** 2026-06-20

**Authors:** Usama Yaseen, Anne Nurbaity, Betty Natalie Fitriatin, Tualar Simarmata, Irham Habibillah Sujudi, Farhan Ahmad, Adellya Safitri

**Affiliations:** 1 Department of Soil Science and Land Resources, Faculty of Agriculture, Padjadjaran University, Jalan Ir. Soekarno Km. 21, Jatinangor, Sumedang, 45363 Indonesia; 2 Department of Agronomy, Faculty of Agriculture, Padjadjaran University, Jalan Ir. Soekarno Km. 21, Jatinangor, Sumedang, 45363 Indonesia

**Keywords:** Organic amendments, Microbial biofertilizers, Nutrient uptake efficiency, Soil microbial activity, Legume productivity

## Abstract

**Background:**

Sustainable intensification of soybean production requires strategies that simultaneously enhance plant growth, nutrient acquisition, and microbial symbiosis, particularly in nutrient-limited soils.

**Methods:**

This experiment investigated the combined effects of organic amendments (Biochar, Vermicompost) and microbial biofertilizers [encapsulated *Rhizobium* and arbuscular mycorrhizal fungi (AMF)] on the morphophysiological performance of black soybean (*Glycine max*).

**Results:**

All treatments, the integration of Vermicompost and Biochar with dual inoculation (encapsulated *Rhizobium* + AMF) consistently produced the most pronounced improvements. Leaf area index and height–diameter ratio were significantly enhanced from 21 days after planting onward, with the strongest canopy expansion and structural growth observed at 28 days. Biomass of shoots and roots production was maximized under Biochar + dual inoculation, surpassing all other treatments. Phosphorus uptake was significantly elevated, and AMF root colonization reached 80%, the highest across treatments. This treatment also supported the greatest nitrogen-fixing bacterial population (7.63 × 105 CFU g⁻1 soil), indicating synergistic microbial interactions. Data analyses confirmed that improvements in morphophysiology, nutrient acquisition, and microbial activity were strongly interrelated, with the majority of variance explained by coordinated responses under the Biochar + dual inoculation system.

**Conclusion:**

The integration of Biochar with encapsulated *Rhizobium* and AMF represents a highly effective strategy to enhance black soybean productivity and microbial symbiosis in Inceptisol, offering a promising pathway toward sustainable crop management.

## Introduction

Global agriculture faces the dual challenge of sustaining crop productivity while mitigating soil degradation and climate change impacts [7]. Legumes, particularly soybean (*Glycine max* L.), are central to food security due to their high protein content and their ability to improve soil fertility through symbiotic nitrogen fixation [1, 27]. Black soybean, a nutritionally enriched variant widely cultivated in Asia, holds promise for diversifying diets and enhancing agroecosystem resilience [33]. Its productivity is constrained in marginal soils such as Inceptisols, which are characterized by low organic matter, poor nutrient retention, and limited biological activity [13]. Conventional reliance on chemical fertilizers has provided short-term yield gains but often exacerbates soil quality decline, underscoring the need for sustainable alternatives [1]. Soil organic amendments soil structure and porosity**,** enhancing aeration and water infiltration [24]. They increase soil organic carbon, which boosts microbial activity and nutrient cycling for sustainable fertility [3]. Organic inputs also enhance nutrient availability, supplying nitrogen, phosphorus, and micronutrients essential for plant growth [24].

Vermicompost and Biochar are effective organic amendments that improve soil physicochemical properties, optimize nutrient dynamics, and stimulate beneficial microbial communities [10]. Vermicompost provides readily available nutrients and bioactive compounds, while Biochar supports long-term carbon sequestration and enhances cation exchange capacity [18]. Organic amendments contribute to carbon sequestration, mitigating climate change while sustaining crop productivity [24]. These amendments also stimulate beneficial microbial activity and suppress soil-borne pathogens, creating a favorable environment for plant growth [3]. Biofertilizers, particularly arbuscular mycorrhizal fungi (AMF) and nitrogen-fixing bacteria (NFB), offer biological solutions to enhance nutrient uptake and symbiotic efficiency [17, 19, 32]. When combined, amendments provide the physical and chemical foundation, while biofertilizers activate biological processes, thereby improving colonization efficiency and stress tolerance in crops [24]. Recent advances in microbial inoculant formulation highlight the importance of carrier materials and encapsulation technologies in improving microbial viability and field performance [11].

The present study aims to investigate the interactive effects of Vermicompost and Biochar, in combination with encapsulated *Rhizobium* and arbuscular mycorrhizal fungi (AMF), on black soybean cultivated in Inceptisols. The study specifically evaluates plant morphophysiological responses (leaf area index and height–diameter ratio), biomass production (shoot and root Biomass and nodulation index), nutrient uptake efficiency (nitrogen and phosphorus uptake), and microbial activity indicators (AMF root colonization and nitrogen-fixing bacterial population). By integrating these functional parameters, this research seeks to clarify how combined organic amendments and microbial inoculants influence plant performance and soil microbial activity, thereby providing a scientific basis for improving sustainable soybean production in marginal Inceptisol environments.

## Material methods

### Experimental site, soil characteristics, and study period

The experiment was conducted from April to November 2025 in a controlled plastic house at Bale Tatanen (≈770 m above sea level). Mean air temperature during the experimental period was 25.15 °C, with minimum and maximum values of 22.84 °C and 27.84 °C, respectively, while mean relative humidity was 84.86% (72.32–98.13%). Rhizobium encapsulation, as well as isolation and physiological characterization, including compatibility and starch hydrolysis tests, were conducted at the Soil Biology Laboratory, Department of Soil Science and Land Resources, Faculty of Agriculture, Padjadjaran University, Indonesia. The experimental soil was a Jatinangor Inceptisol. The physicochemical properties of the soil used in the experiment, analyzed at the Soil Chemistry and Plant Nutrition Laboratory, Padjadjaran University, are presented in Table 1, including soil pH, nutrient status, exchangeable cations, cation exchange capacity, and AMF spore abundance.

**Table 1:** Physicochemical properties of the experimental soil

Parameter	Unit	Value	Classification
pH (H₂O)	–	5.7	Slightly acidic
pH (KCl)	–	6.34	–
Organic C	%	2.49	Moderate
Total N	%	0.35	Moderate
C/N ratio	–	7	Low
P₂O₅ (HCl 25%)	mg 100 g⁻1	35.09	Moderate
Available P₂O₅ (Bray I)	ppm P	8.83	Low
K₂O (HCl 25%)	mg 100 g⁻1	16.88	Low
Exchangeable K	cmol kg⁻1	0.34	Low
Exchangeable Na	cmol kg⁻1	0.18	Low
Exchangeable Ca	cmol kg⁻1	10.61	High
Exchangeable Mg	cmol kg⁻1	3.94	High
Cation exchange capacity (CEC)	cmol kg⁻1	24.81	Moderate
Exchangeable Al	cmol kg⁻1	0.19	–
Exchangeable H	cmol kg⁻1	0.34	–
Al saturation	%	1.23	Low
AMF spore number	spores per 25 g soil	377	–
Base saturation	%	60.76	Moderate

### Instruments and reagents

Plant growth and physiological measurements were conducted using a SPAD chlorophyll meter for chlorophyll assessment, a digital caliper and ruler for plant height and stem diameter measurements, and a laboratory drying oven for biomass determination. Leaf area measurements were obtained using calibrated leaf area tracing charts.

Rhizobium isolates used for bioencapsulation consisted of strains BJ-H6, BJ-K3, and BJ-K4 [13] and Arbuscular mycorrhizal fungi (AMF) inoculum consisted of a mixed culture of Glomus spp. and Gigaspora spp. obtained from the Soil Biology Laboratory, Department of Soil Science and Land Resources, Faculty of Agriculture, Padjadjaran University. Bioencapsulated Rhizobium inoculants were formulated using sodium alginate (≥ 99% purity, Merck), starch (analytical grade, ≥ 99% purity, Sigma-Aldrich), and kaolin (analytical grade, ≥ 98% purity, Merck) as carrier materials, while calcium chloride (CaCl₂; ≥ 99% purity, Merck) was used as the cross-linking agent. AMF inoculum was applied at 15 g plant⁻1, whereas bioencapsulated nitrogen-fixing Rhizobium was applied at 1 g per polybag (10⁷ CFU ml⁻1). The composition of the bioencapsulated Rhizobium inoculant used in this study is summarized in Table 2.

**Table 2:** Composition of bioencapsulated *Rhizobium* inoculant used in the experiment

Component	Concentration/Specification	Function
*Rhizobium* culture	Bacterial suspension	Biofertilizer inoculum
Sodium alginate	Sodium alginate	Encapsulation matrix
Starch	4% (w/v)	Nutrient source and stabilizer
Kaolin	1% (w/v)	Structural support material
Calcium chloride (CaCl₂)	0.1 M	Cross-linking agent

Biochar used in this study was a commercial organic soil amendment product (Biotree 3 in 1, Fellagro) composed of charcoal (biochar), dolomite, and liquid organic fertilizer. According to the manufacturer’s laboratory analysis, the product contained 52.5% organic C, pH 8.48, 0.93% nitrogen, 252 ppm available Fe, and 32.65 ppm Zn. The product was also reported to be free from pathogenic contaminants. The vermicompost used in this experiment was a commercial product (Verminat Plus Vermicompost, Kebon Kita). Chemical and biological analysis indicated that the vermicompost had a pH of 7.86 and water content of 26.89%. The material contained 2.13% K₂O, 3.86% P₂O₅, 1.85% total nitrogen, and 27.98% organic C, with a C/N ratio of 15.12. In addition, the vermicompost contained several micronutrients, including 5.14% magnesium, 121.19 ppm zinc (Zn), 242.7 ppm boron (B), and 59.23 ppm copper (Cu).

AMF inoculation was conducted twice: once 1 week before planting and again 2 weeks after planting. The pre-planting application involved sprinkling the inoculant directly into the planting hole, while the second application was applied around the root zone to ensure effective colonization. Baseline fertilization was standardized across treatments using urea (0.31 g plant⁻^1^), triple superphosphate (0.94 g plant⁻^1^), and potassium chloride (0.63 g plant⁻^1^). Follow-up fertilization was conducted at 7 days after sowing (DAS) by placing the fertilizers into a hole 10 cm from the plant base, and again at 21 DAS by broadcasting the same dosages evenly around the plants while maintaining a 10 cm distance to optimize nutrient uptake.

### Experimental design and procedure

The experiment was conducted using a randomized complete block design (RCBD) with a single factor comprising 12 treatment combinations derived from different organic amendments and microbial biofertilizer applications, with 3 replicates per treatment, yielding a total of 36 experimental pots. Each experimental unit consisted of a polybag (35 × 35 cm) filled with 5 kg of soil. Two plants were initially grown per pot, and one week after emergence, they were thinned to retain one healthy plant per pot. Vermicompost and Biochar were each applied at 62.5 g per plant, equivalent to 5 t ha⁻^1^. Treatment combinations are shown in Table 3.

**Table 3:** Treatment combinations for soil organic amendments and biofertilizer applications

Treatment Code	Soil Organic Amendment	Biofertilizer Treatment
O0B0	None	No biofertilizer
O0B1	None	AMF
O0B2	None	Encapsulated Rhizobium
O0B3	None	Encapsulated Rhizobium + AMF
O1B0	Vermicompost	No biofertilizer
O1B1	Vermicompost	AMF
O1B2	Vermicompost	Encapsulated Rhizobium
O1B3	Vermicompost	Encapsulated Rhizobium + AMF
O2B0	Biochar	No biofertilizer
O2B1	Biochar	AMF
O2B2	Biochar	Encapsulated Rhizobium
O2B3	Biochar	Encapsulated Rhizobium + AMF

### Observed parameters

#### Plant morphological and physiological traits

Leaf area index was determined Traditional Manual Method (Gao et al., 2024) recording the number of leaves per plant (NL). Leaf area (AL) was measured by tracing fully expanded leaves on chart paper and calculating the area in cm^2^. The ground area occupied by each plant was recorded as AG (cm^2^). Leaf area index was calculated as:

$$\mathrm{Leaf}\;\mathrm{Area}\;\mathrm{Index}=\left(\frac{\mathrm{NL}\times\;\mathrm{AL}}{\mathrm{AG}}\right)$$

The height-to-diameter ratio was determined to evaluate plant structural growth. Plant height was recorded as H, and stem diameter was measured as D (Thornley, 1999). Height to diameter ratio was calculated as:

$$\mathrm{Height-to-diameter}\;\mathrm{ratio}=\left(\frac{\mathrm{Plant}\;\mathrm{Height}}{\mathrm{Plant}\mathrm{stem}\;\mathrm{Diameter}}\right)$$

Shoot and root biomass were calculated to assess their efficiency. Shoot dry weight was recorded as DWₛ, and root dry weight was recorded as DWᵣ.

$$\mathrm{Biomass}\;\mathrm{of}\;\mathrm{shoots}\mathrm{and}\;\mathrm{roots}\;\mathrm{production}=\left(\frac{\mathrm{DWs}+\mathrm{DWr}}{2|}\right)$$

The normalized mean nodulation index was calculated to evaluate nodulation efficiency. Effective nodules were recorded as Neff, total nodules as Ntotal, effective nodule weight as Weff, and total nodule weight as Wtotal (Herridge et al., 2008). Normalized mean nodulation index was calculated as:

$$\mathrm{Normalized}\;\mathrm{mean}\;\mathrm{nodulation}\;\mathrm{index}=\left(\frac{\mathrm{Neff}}{\mathrm{Normal}}+\frac{\mathrm{Weff}}{\mathrm{Wtotal}}\right)\times 100$$

#### Nutrient measurements

Nitrogen uptake was determined using the Kjeldahl digestion method. Oven-dried and ground plant samples were digested, and total nitrogen concentration was measured. Nitrogen uptake was calculated by multiplying nitrogen concentration by plant dry weight (Kaur et al., 2023). Phosphorus uptake was determined using the wet ashing method. Plant samples were digested using acid oxidation, and phosphorus concentration was quantified colorimetrically. Phosphorus uptake was calculated by multiplying phosphorus concentration by plant dry weight (Pang et al., 2024).

#### Microbial parameters

The nitrogen-fixing bacterial population was determined using the total plate count method. Serial dilutions of soil samples were prepared and plated on the appropriate growth medium (Thuy & Tuu, 2025). After incubation, visible colonies were counted and expressed as colony-forming units per gram of soil (CFU g⁻^1^). AMF root colonization was assessed using the root clearing and staining method followed by microscopic examination after harvest (Jarratt-Barnham et al., 2024). One gram of fine root samples was collected, washed, cleared, and stained to visualize fungal structures. Ten root segments were randomly selected and examined under a light microscope. The percentage of colonized root segments was calculated as the AMF root colonization index.

### Data analysis

The experimental data were analyzed using an analysis of variance appropriate for a randomized complete block design with a single factor comprising 12 treatment combinations**.** Treatment means were compared using Tukey’s HSD post hoc test at a significance level of p ≤ 0.05. Prior to analysis, assumptions of normality and homogeneity of variance were verified to ensure statistical validity. All statistical computations and graphical visualizations were performed using OriginLab (OriginPro v.2025) and SmartPLS (SmartPLS v.4), which provided advanced tools for data modeling and multivariate analysis. Results are presented as mean ± standard error, with graphical outputs illustrating treatment effects across growth, microbial, and physiological parameters.

## Results

### Effects of organic amendments and biofertilizers on plant growth, nutrient uptake, and microbial activity

The analysis of variance revealed that treatment effects on Leaf Area Index (LAI) varied across observation times (Table 4). At 14 days after planting (DAP), LAI was not significantly affected by treatments (p > 0.05). Significant (*p < *0.05) soil organic amendment with biofertilizer *(AMF* and *Rhizobium)* treatment effects were detected from 21 DAP onward, with p-values of 0.039 at 21 DAP, 0.001 at 28 DAP, 0.009 at 35 DAP, 0.007 at 42 DAP, and 0.003 at 48 DAP. The lowest p-value was observed at 28 DAP, indicating the strongest soil organic amendment effect with the biofertilizer treatment at this stage. The height–diameter ratio (HDR) showed no significant (p > 0.05) treatment effect at 14 DAP (*p* = 0.462) and 21 DAP (*p* = 0.061). Significant (*p < *0.05) differences among treatments emerged from 28 DAP onward, with p-values of 0.024 at 28 DAP, 0.026 at 35 DAP, 0.043 at 42 DAP, and 0.034 at 48 DAP.

**Table 4:** Mean values and significance levels (*p*-values) for Leaf Area Index, Height–diameter ratio, and biological/microbial variables under soil organic amendment (Vermicompost and Biochar) with biofertilizer *(AMF* and *Rhizobium)* treatment

Parameters	14 Days	21 Days	28 Days	35 Days	42 Days	48 Days
Leaf Area Index	0.563	0.039*	0.001*	0.009*	0.007*	0.003*
Height-Diameter Ratio	0.462	0.061	0.024*	0.026*	0.043*	0.034*
Plant Growth, Nutrient Acquisition, and Microbial Interactions	Normalized Mean Nodulation Index (%)	NFB population (10^5)	N uptake (mg plant^−1^)	P uptake (mg plant^−1^)	Biomass of shoots and roots(g plant^−1^)	AMF root colonization (%)
	0.137	0.059	0.103	0.003*	0.000**	0.000**

The normalized mean nodulation index was not significantly affected by organic amendments and biofertilizer treatments (*p* = 0.137). Similarly, the NFB population did not show a significant response to treatments (*p* = 0.059). Nitrogen uptake also did not differ significantly among treatments (*p* = 0.103). Significant effects of organic amendments and biofertilizer treatments on P uptake were observed (*p* = 0.005). The Biomass of shoot and root production was significantly (*p < *0.05) affected (*p* = 0.000), indicating strong variation among organic amendments and biofertilizer treatments. The strongest organic amendments and biofertilizer treatment response among microbial parameters was recorded for AMF root colonization, which was highly significant (*p* = 0.000).

### Morphophysiological responses

**Fig. 1:**
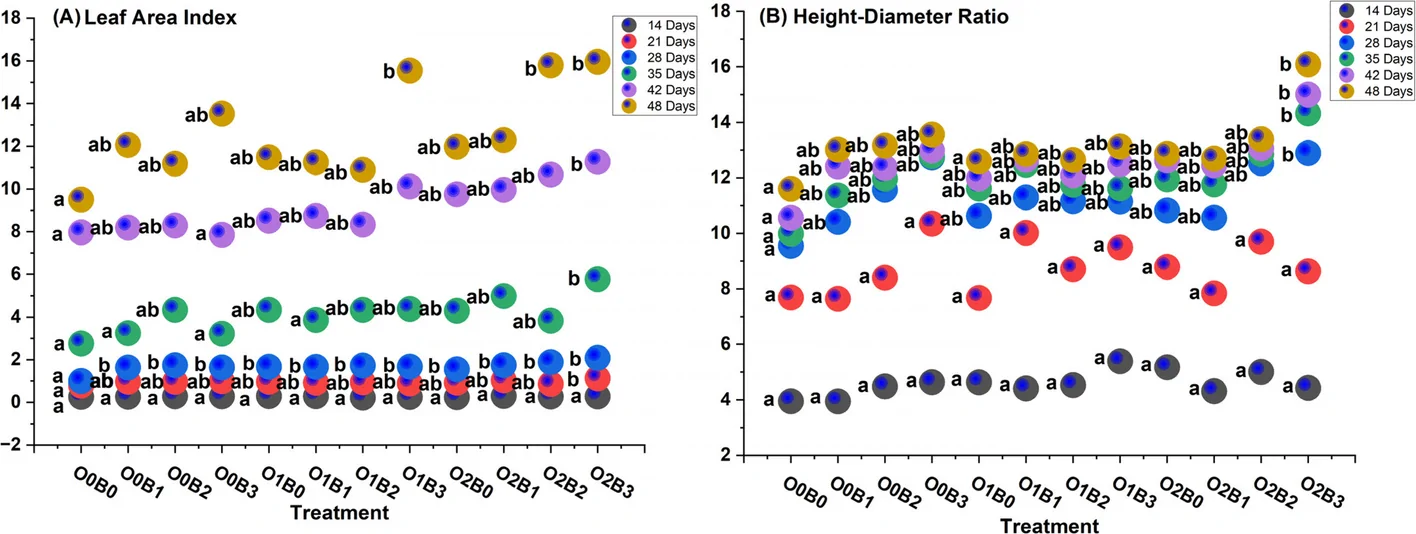
**A** and **B** Effects of soil organic amendments and biofertilizer treatments on leaf area index and height–diameter ratio measured. Soil organic amendments: O0 (no amendment), O1 (Vermicompost), O2 (Biochar); biofertilizers: B0 (none), B1 (Rhizobium), B2 (AMF), B3 (Rhizobium + AMF)

Leaf Area Index (LAI) progressively increased with plant age across all treatments. At 14 days, LAI values were low and did not differ significantly among treatments (Fig. 1A). From 21 days onward, numerical differences became more evident, although statistical separation remained limited during early growth stages. At 28 days, treatments integrating Biochar with encapsulated Rhizobium + AMF and Vermicompost with encapsulated Rhizobium + AMF exhibited higher LAI values than the non-amended, non-biofertilized control, with clear statistical differences. This trend continued at 35 and 42 days, with combined organic amendment and dual biofertilizer treatments consistently producing greater canopy development than single inoculations or amendment-only treatments. At 48 days, the highest LAI values were observed in the Biochar combined with encapsulated Rhizobium + AMF treatment, followed closely by the Vermicompost combined with encapsulated Rhizobium + AMF and the Biochar combined with encapsulated Rhizobium treatments. In contrast, treatments without biofertilizer application generally showed lower LAI values at all growth stages. Standard deviation bars indicated moderate variability among replicates, with slightly greater dispersion in treatments combining organic amendments and biofertilizers during later growth stages.

The height–diameter ratio (HDR) increased gradually over time in all treatments (Fig. 1B). At 14 and 21 days, differences among treatments were minimal and largely nonsignificant. From 28 days onward, clearer separation among treatments was observed. The highest HDR values were consistently recorded in the treatment combining Biochar with encapsulated Rhizobium + AMF, particularly at 35, 42, and 48 days, where it formed a distinct statistical group compared to several other treatments. Treatments combining Vermicompost with encapsulated Rhizobium + AMF and Biochar with encapsulated Rhizobium also showed comparatively elevated HDR values at later growth stages. Single inoculations and organic amendments without biofertilizers yielded intermediate responses, whereas the non-amended, non-biofertilized control maintained lower HDR values throughout the experimental period. Standard deviation bars demonstrated acceptable replicate consistency, although variability increased slightly at later growth stages under combined treatment applications.

### Plant biomass production

**Fig. 2:**
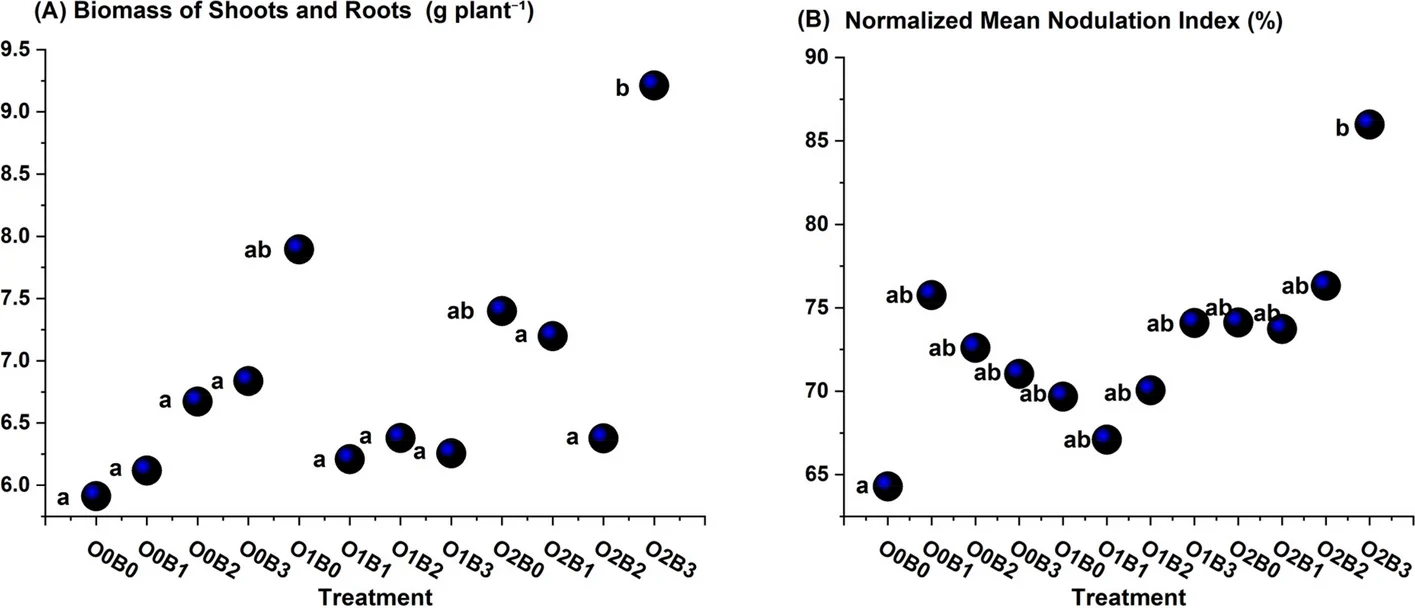
**A** and **B** Effects of soil organic amendments and biofertilizer treatments on Biomass of shoots and roots production (g plant⁻.^1^) and normalized mean nodulation index (%). Soil organic amendments: O0 (no amendment), O1 (Vermicompost), O2 (Biochar); biofertilizers: B0 (none), B1 (Rhizobium), B2 (AMF), B3 (Rhizobium + AMF)

Biomass of shoots and roots production differed significantly among treatments (Fig. 2A). The highest mean shoot and root biomass was recorded in the treatment combining Biochar with encapsulated Rhizobium + AMF**,** which was statistically superior to most other treatments, as indicated by distinct significance lettering. Treatments integrating organic amendments with biofertilizers generally exhibited greater shoot and root biomass than their respective non-biofertilized controls. Vermicompost combined with encapsulated Rhizobium + AMF and Biochar combined with encapsulated Rhizobium showed comparatively elevated values, though not always significantly different from intermediate groups. Treatments without biofertilizer application, including no amendment, Vermicompost**,** and Biochar, exhibited relatively lower mean values. Standard deviation bars indicated moderate variability among replicates, with greater dispersion observed in some combined treatments, whereas control treatments generally showed narrower variability.

The normalized mean nodulation index varied among treatments, although the overall treatment effect was not statistically significant at the defined probability threshold (Fig. 2B). Numerically, the highest nodulation index was observed in the treatment combining Biochar with encapsulated Rhizobium + AMF, followed by treatments integrating Vermicompost or Biochar with individual or combined biofertilizers. Treatments receiving only organic amendments without biofertilizers demonstrated comparatively lower nodulation indices. The no-amendment treatment with no biofertilizer showed one of the lowest mean values.

### Plant nutrient acquisition

**Fig. 3:**
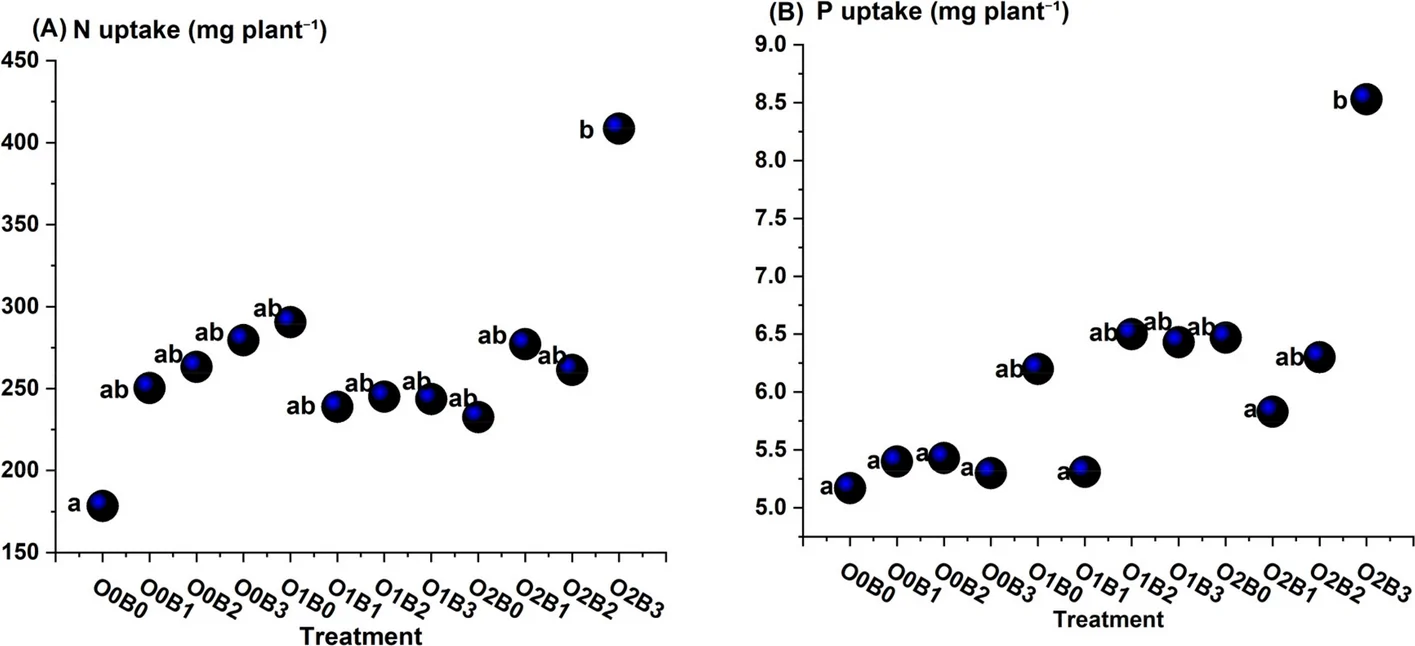
**A** and **B** Effects of soil organic amendments and biofertilizer treatments on nitrogen uptake (mg plant⁻^1^) and phosphorus uptake (mg plant⁻.^1^). Soil organic amendments: O0 (no amendment), O1 (Vermicompost), O2 (Biochar); biofertilizers: B0 (none), B1 (Rhizobium), B2 (AMF), B3 (Rhizobium + AMF)

Nitrogen uptake varied among treatments; however, the overall treatment effect was not statistically significant at the defined probability level (Fig. 3A). The highest nitrogen uptake was observed in the treatment combining Biochar with encapsulated Rhizobium + AMF, which formed a distinct statistical grouping in pairwise comparison. Treatments integrating Vermicompost or Biochar with individual biofertilizers showed intermediate nitrogen uptake values, whereas the non-amended, non-biofertilized control recorded comparatively lower values. Organic amendments without biofertilizer application also exhibited moderate nitrogen uptake. Standard deviation bars indicated noticeable variability among replicates, particularly in treatments combining organic amendments with dual biofertilizers, where dispersion was greater compared to control treatments.

Phosphorus uptake was significantly influenced by treatments (Fig. 3B). The highest phosphorus uptake was recorded in the treatment combining Biochar with encapsulated Rhizobium + AMF, which was statistically superior to several other treatments. Treatments involving Vermicompost or Biochar combined with individual biofertilizers also exhibited relatively elevated phosphorus uptake compared to the non-amended, non-biofertilizer control. Treatments without biofertilizer and organic amendment application generally showed lower phosphorus uptake values.

### Soil microbial activity

**Fig. 4:**
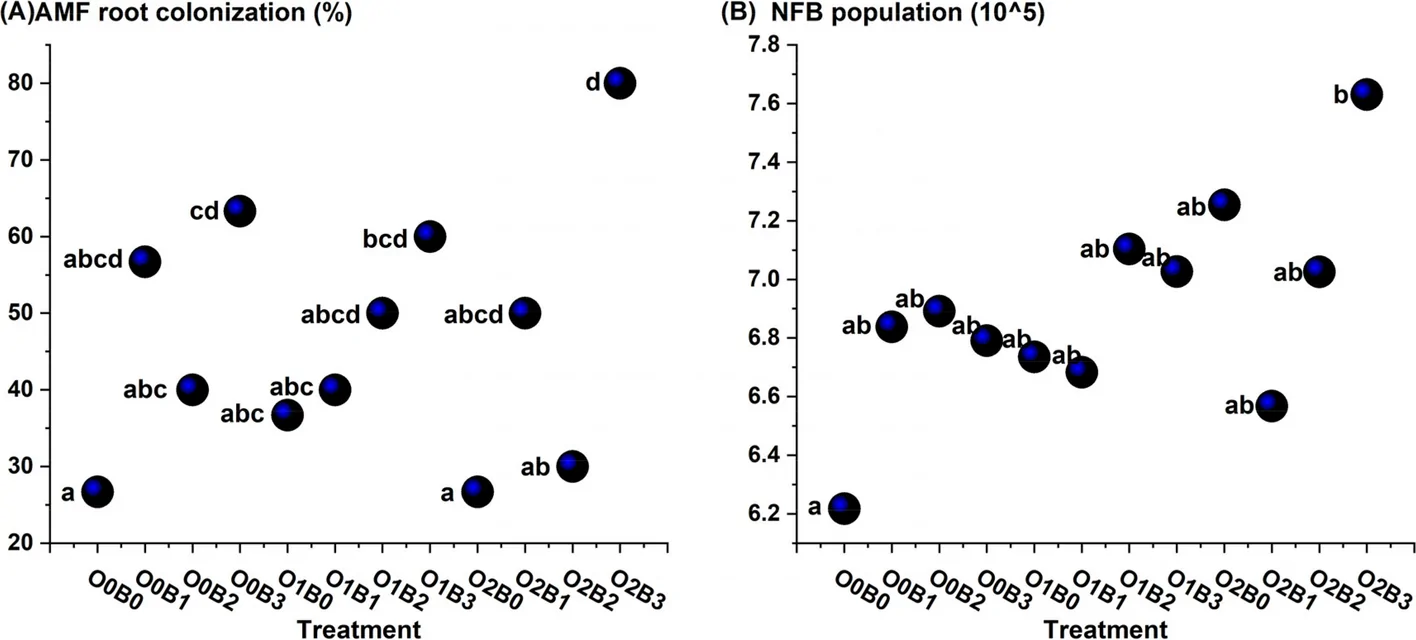
**A** and **B** Effects of soil organic amendments and biofertilizer treatments on arbuscular mycorrhizal fungi colonization and nitrogen-fixing bacterial population. Soil organic amendments: O0 (no amendment), O1 (Vermicompost), O2 (Biochar); biofertilizers: B0 (none), B1 (Rhizobium), B2 (AMF), B3 (Rhizobium + AMF)

Arbuscular mycorrhizal fungi colonization differed significantly among treatments, ranging from 26 to 80% (Fig. 4A). The highest colonization (80%) was observed with Biochar and Encapsulated Rhizobium and AMF, with a standard deviation of 14%. This was followed by None with Encapsulated Rhizobium and AMF at 63% (standard deviation 5%) and Biochar with AMF at 60% (standard deviation 8%). Moderate colonization levels were recorded under Vermicompost with Encapsulated Rhizobium and AMF at 57% (standard deviation 17%), Vermicompost with Encapsulated Rhizobium at 50% (standard deviation 8%), and Vermicompost with AMF at 40% (standard deviation 8%). Lower colonization was observed in Vermicompost without biofertilizer at 37% (standard deviation 12%) and Biochar with Encapsulated Rhizobium at 30% (standard deviation 8%). The minimum colonization values were recorded under Biochar without biofertilizer at 27% (standard deviation 5%) and None without biofertilizer at 26% (standard deviation 5%). Treatments receiving combined Encapsulated Rhizobium and AMF consistently exhibited higher colonization percentages compared with single inoculation or non-inoculated treatments. Greater variability was evident in treatments containing Vermicompost combined with biofertilizers.

Nitrogen-fixing bacterial population ranged from 6.20 to 7.63 × 10^5^ CFU g⁻^1^ soil across treatments. The highest population, 7.63 × 10^5^ CFU g⁻^1^ soil, was observed under Biochar with Encapsulated Rhizobium and AMF, with a standard deviation of 0.05 × 10^5^. Vermicompost followed this with Encapsulated Rhizobium and AMF at 7.02 × 10^5^ (standard deviation 0.11 × 10^5^) and None with AMF at 7.10 × 10^5^ (standard deviation 0.55 × 10^5^). Intermediate populations were recorded under Biochar with Encapsulated Rhizobium at 7.03 × 10^5^ (standard deviation 0.11 × 10^5^), Biochar with AMF at 6.89 × 10^5^ (standard deviation 0.29 × 10^5^), and Vermicompost with Encapsulated Rhizobium at 6.84 × 10^5^ (standard deviation 0.49 × 10^5^). Lower values were observed in Biochar without biofertilizer at 6.73 × 10^5^ (standard deviation 0.39 × 10^5^), while the lowest population, 6.20 × 10^5^ CFU g⁻^1^ soil, was recorded under None without biofertilizer, with a standard deviation of 0.05 × 10^5^. Treatments combining Encapsulated Rhizobium and AMF under biochar amendment showed comparatively lower variability and consistently higher bacterial counts.

### Correlation analysis

**Fig. 5:**
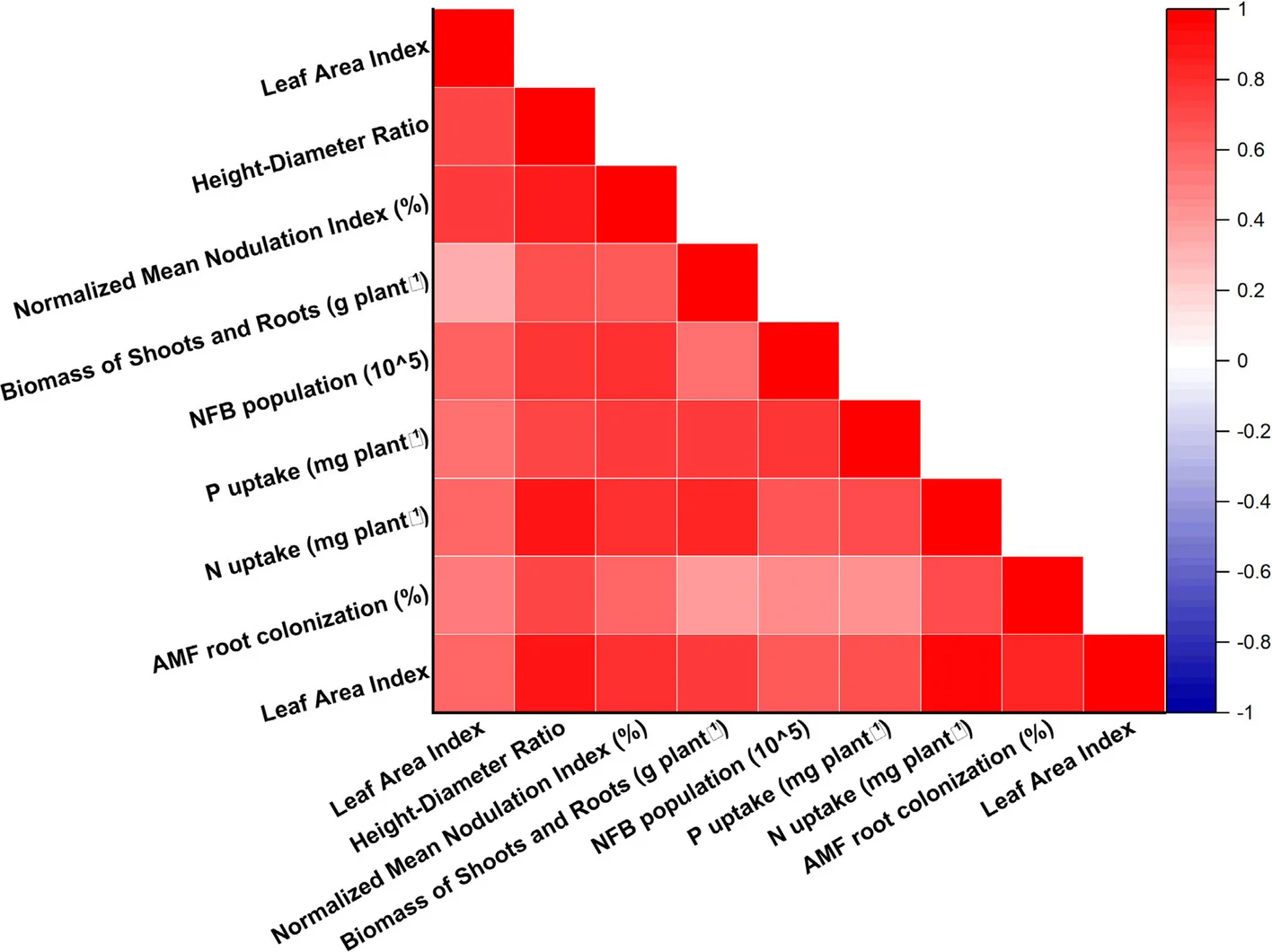
Pearson correlation matrix among the studied parameters. Color intensity represents the strength and direction of Pearson correlation coefficients, ranging from − 1 (strong negative correlation) to + 1 (strong positive correlation)

The correlation matrix revealed predominantly strong positive associations among the studied growth, physiological, microbial, and nutrient parameters (Fig. 5). Leaf area index showed strong positive correlations with height-to-diameter ratio, normalized mean nodulation index, shoot and root biomass production, nitrogen-fixing bacterial population, phosphorus uptake, nitrogen uptake, and AMF root colonization. The height-to-diameter ratio was also strongly and positively correlated with the normalized mean nodulation index, shoot and root biomass production, nitrogen-fixing bacterial population, phosphorus uptake, nitrogen uptake, and AMF root colonization.

The normalized mean nodulation index exhibited a strong positive relationship with shoot and root biomass production, nitrogen-fixing bacterial population, phosphorus uptake, nitrogen uptake, and AMF root colonization. Biomass production of shoots and roots was positively correlated with nitrogen-fixing bacterial population, phosphorus uptake, nitrogen uptake, and AMF root colonization, with intensities ranging from moderate to strong relative to structural growth traits. The nitrogen-fixing bacterial population demonstrated strong positive correlations with phosphorus and nitrogen uptake and a positive association with AMF root colonization. Phosphorus uptake showed a strong positive correlation with nitrogen uptake and a positive association with AMF root colonization. Nitrogen uptake was also positively correlated with AMF root colonization. No negative correlations were observed among the measured parameters, indicating a consistent positive interrelationship across plant growth, microbial activity, and nutrient acquisition variables.

### Principal component analysis

**Fig. 6:**
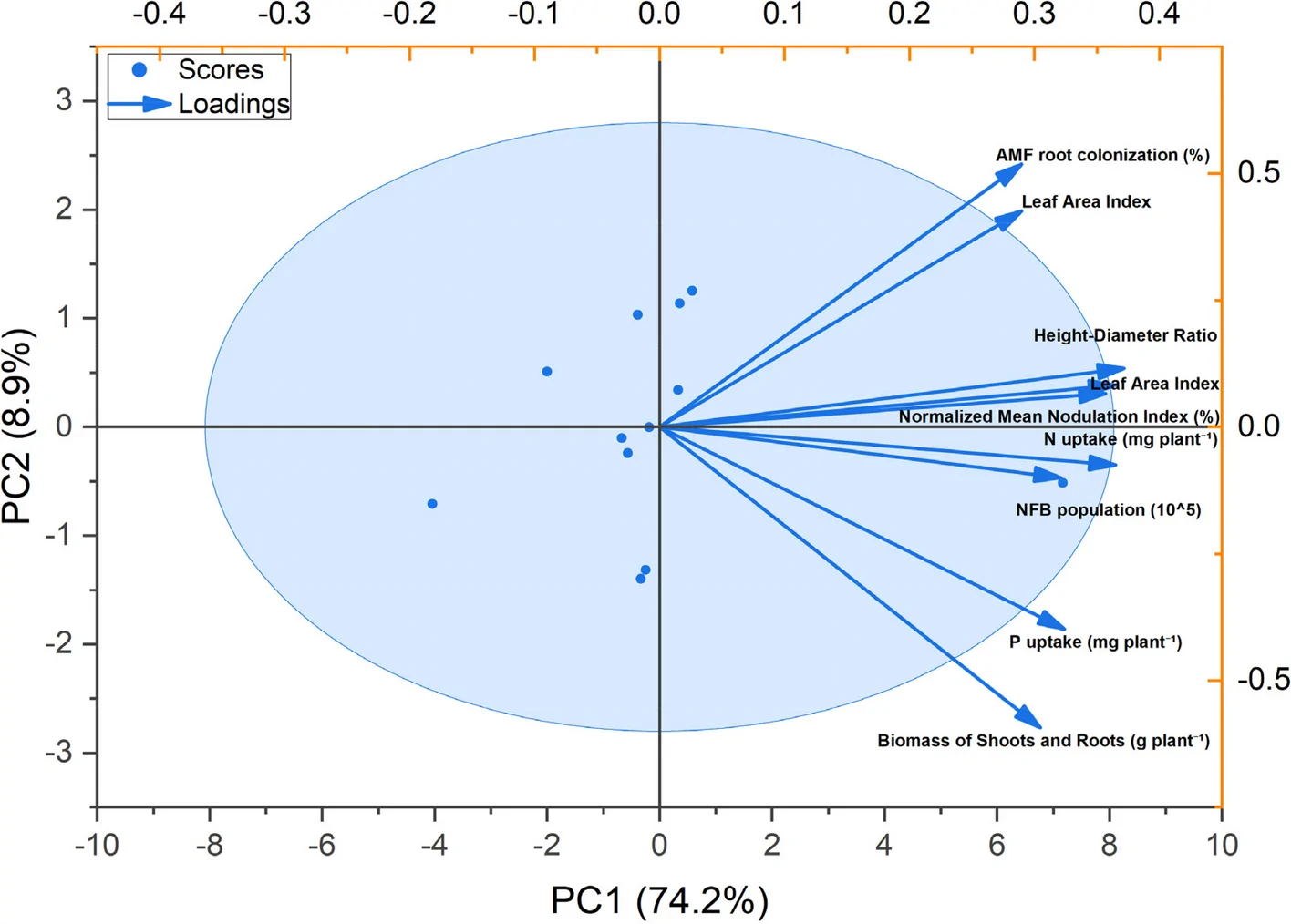
Principal component analysis biplot showing scores and loadings of the studied parameters

Principal component analysis explained 83.1% of the total variance, with PC1 accounting for 74.2% and PC2 accounting for 8.9% (Fig. 6). All measured parameters exhibited positive loadings along PC1, indicating a common directional contribution to overall variability. Leaf area index and AMF root colonization showed strong positive loadings on PC1 and clear positive contributions to PC2. Height-to-diameter ratio and the normalized mean nodulation index were positively associated with PC1, with minimal contribution to PC2.

The nitrogen-fixing bacterial population and nitrogen uptake were strongly aligned along the positive axis of PC1, with negligible influence from PC2. Phosphorus uptake and shoot and root biomass production displayed strong positive loadings on PC1 but were negatively associated with PC2, indicating differentiation along PC2. The distribution of treatment scores was primarily structured along PC1, indicating that coordinated changes in growth attributes, microbial activity, and nutrient uptake parameters accounted for the majority of the variability among treatments.

**Fig.7:**
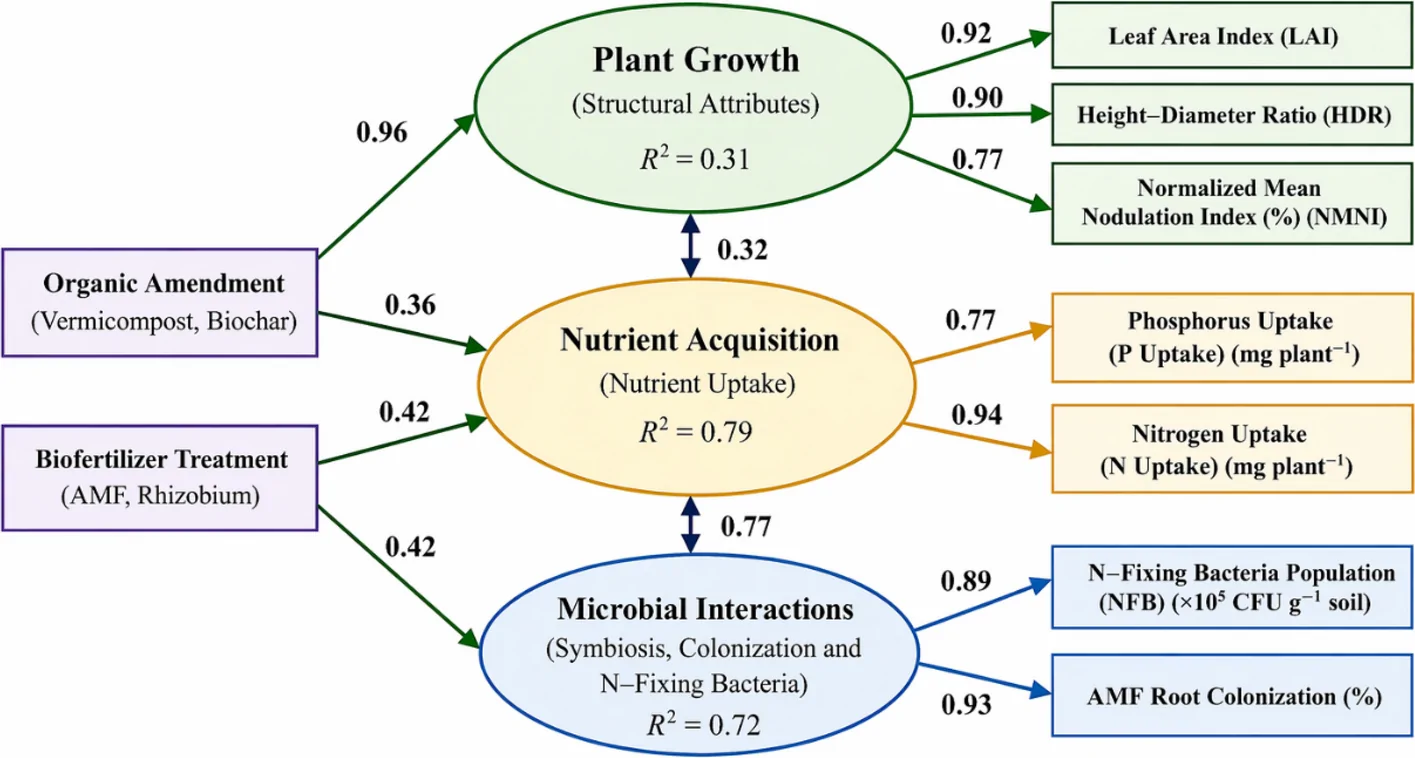
Structural equation modeling (SEM) illustrates the relationships among organic amendment, biofertilizer treatment, plant growth attributes, nutrient acquisition, and microbial interactions. Standardized path coefficients are shown on arrows, and R2 values indicate the variance explained in each endogenous latent construct

The SEM demonstrates that organic amendment and biofertilizer treatment exert direct and indirect influences on plant growth, nutrient acquisition, and microbial interactions. Plant growth is primarily represented by leaf area index, height-diameter ratio, and normalized mean nodulation index, while nutrient acquisition is explained through nitrogen and phosphorus uptake (Fig. 7). Microbial interactions are characterized by AMF root colonization and a nitrogen-fixing bacterial population. Strong path coefficients and high R^2^ values for nutrient acquisition and microbial interactions indicate that improvements in microbial activity substantially enhance nutrient uptake, which, in turn, contributes to improved structural plant growth. The model highlights the integrated role of soil amendments and biofertilizers in regulating plant performance through interconnected biological and physiological processes.

**Fig.8:**
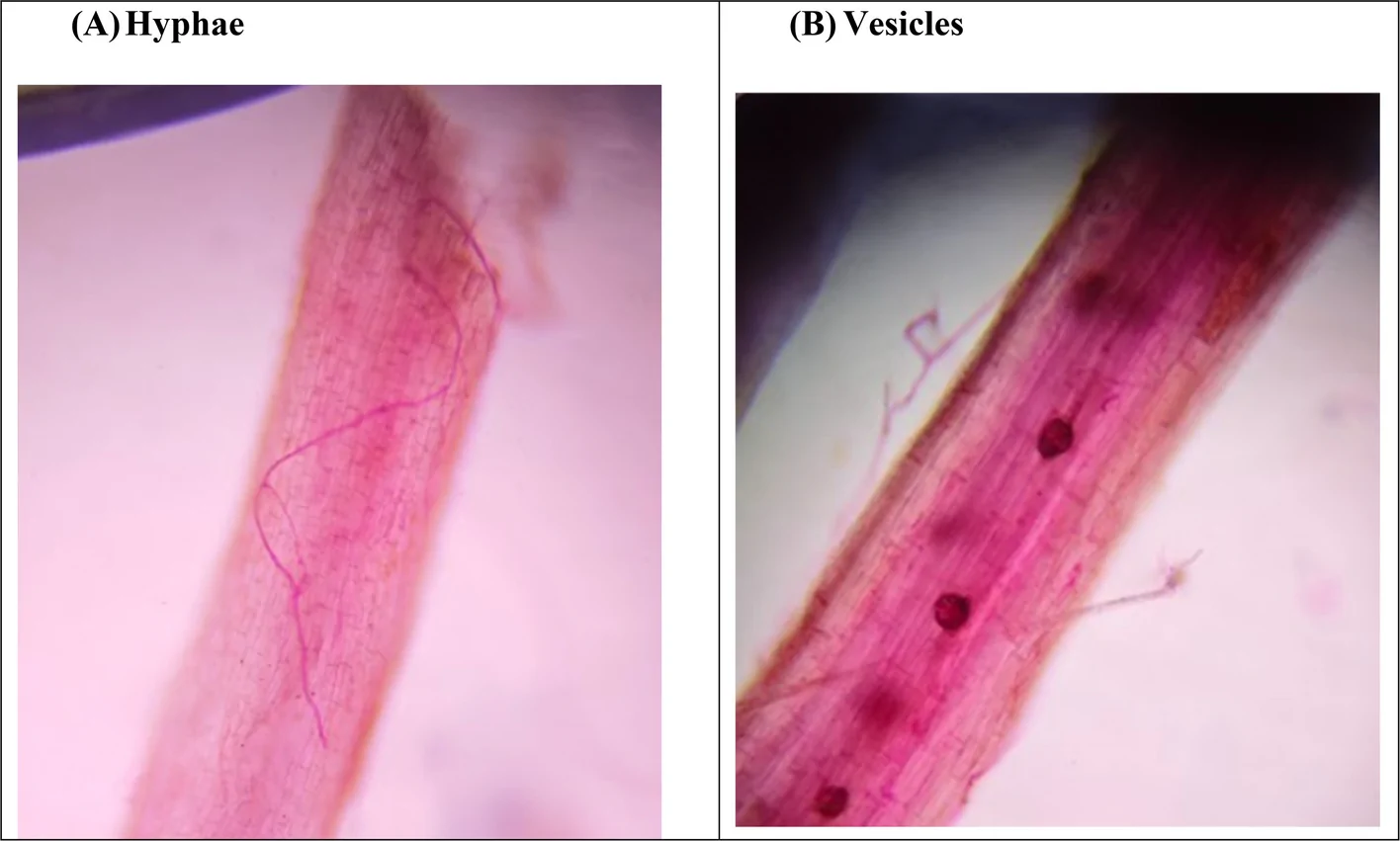
Microscopic observation of arbuscular mycorrhizal fungi (AMF) structures in black soybean roots: (**A**) hyphae and (**B**) vesicles

Microscopic examination of black soybean roots confirmed the presence of characteristic AMF structures, including hyphae and vesicles. These observations provide direct visual evidence of fungal colonization and complement the quantitative data presented. Representative images of each structure are shown (Fig. 8).

**Fig. 9:**
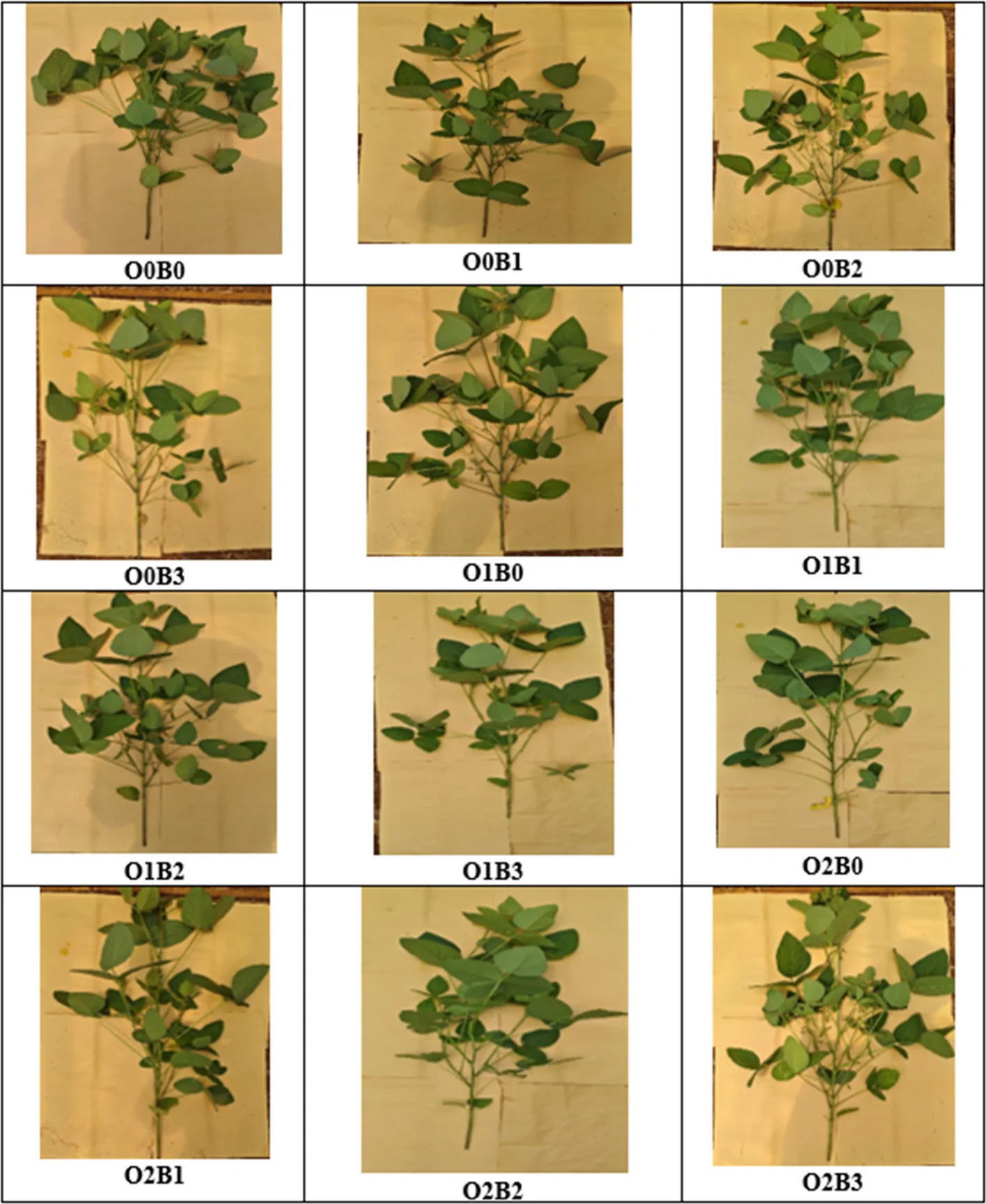
Shoot display of black soybean plants under soil organic amendments: (O0 no amendment, O1 vermicompost, O2 biochar) and biofertilizers: (B0 none, B1 Rhizobium, B2 AMF, B3 Rhizobium plus AMF)

Effects of soil organic amendments (O0 no amendment, O1 vermicompost, O2 biochar) and biofertilizer treatments (B0 none, B1 Rhizobium, B2 AMF, B3 Rhizobium plus AMF) on the shoot display of black soybean plants are shown in (Fig. 9).

**Fig. 10:**
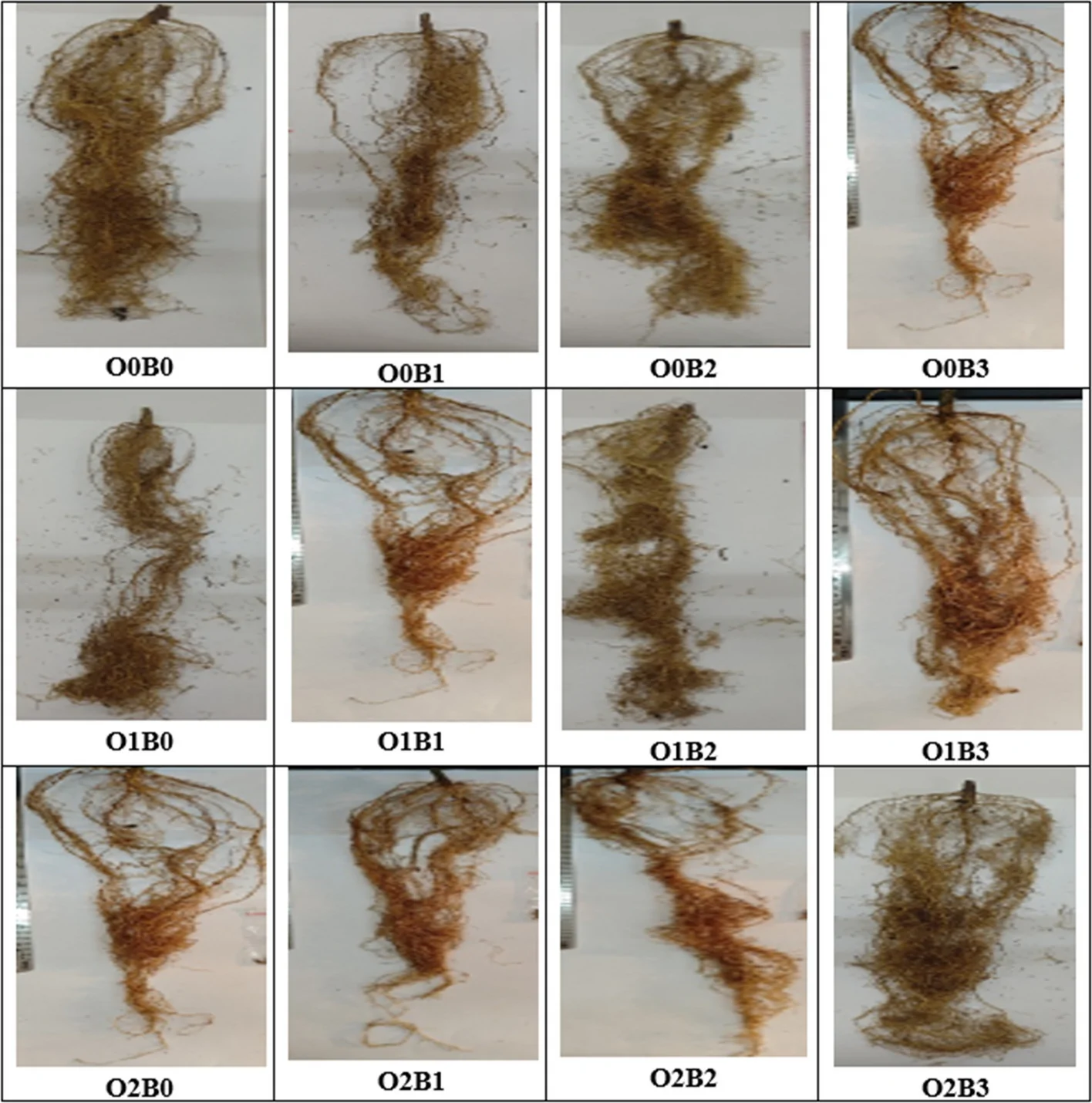
Root display of black soybean plants under soil organic amendments: (O0 no amendment, O1 vermicompost, O2 biochar) and biofertilizers: (B0 none, B1 Rhizobium, B2 AMF, B3 Rhizobium plus AMF)

The dry root appearance of black soybean plants across soil organic amendments (O0 no amendment, O1 vermicompost, O2 biochar) and biofertilizer treatments (B0 none, B1 Rhizobium, B2 AMF, B3 Rhizobium plus AMF), illustrating visual differences in root development among treatments, is shown in (Fig. 10).

## Discussion

An increase in leaf area index reflects progressive canopy expansion and enhanced vegetative development as plant growth advances [29]. Increases in leaf area development reflect improved canopy expansion, likely driven by enhanced nutrient acquisition and microbial-mediated stimulation of vegetative growth, reflecting enhanced canopy development and improved light interception [12]. Biochar’s porous structure and high cation exchange capacity likely improved root aeration and nutrient retention [4]. *AMF* and *Rhizobium* synergistically enhanced nutrient uptake [5] and regulated hormonal signaling [21, 35]. Reduced leaf area development may reflect limitations in nutrient availability and suboptimal physiological support for canopy expansion [20]. Combined organic and microbial inputs significantly enhance leaf area and shoot and root surface biomass in soybean [35].

Increasing height–diameter relationships reflect sustained vegetative elongation and stem structural development [30]. The observed optimization of height–diameter ratios may be explained by enhanced nutrient availability coupled with microbial-mediated regulation of vegetative growth and stem reinforcement [16]. Biochar appears to enhance vertical growth and structural stability, likely by improving nutrient availability and intensifying microbial colonization, thereby reinforcing vegetative development through synergistic interactions [2, 17, 19]. Biochar is attributed to microbial symbiosis, which improves nutrient availability and reinforces vegetative development through synergistic soil plant interactions [9].

Biochar-based treatments promote greater root investment, likely through improved soil aeration and stimulation of microbial activity [31]. Maintaining comparable biomass levels indicates balanced carbon fixation and allocation under the prevailing environmental conditions [23]. Alterations in root-to-shoot balance likely reflect adaptive carbon allocation toward root development, promoted by improved soil physical properties and microbial facilitation of nutrient uptake [15]. Enhanced root development under Biochar likely facilitates nutrient uptake and drought resilience, thereby supporting long-term productivity [35]. Constraints in root-to-shoot balance underscore the regulatory roles of soil amendments and microbial symbiosis in biomass partitioning, consistent with reports that biochar and arbuscular mycorrhizal fungi synergistically enhance root growth and nutrient acquisition, thereby improving root architecture and carbon allocation in soybean systems [14, 22].

The limited response of nodulation and nitrogen-fixing bacterial activity to the applied treatments may be attributed to the presence of indigenous rhizobial populations in the soil, which can reduce the effectiveness of additional inoculation [6]. The availability of soil nitrogen may suppress nodulation, as elevated nitrogen levels are known to inhibit symbiotic nitrogen fixation in legumes. These factors suggest that under the given soil conditions, the effectiveness of biofertilizer application on nodulation-related parameters may be constrained [25]. Co-application of AMF with soil organic amendments has been shown to significantly enhance AMF root colonization and increase plant phosphorus uptake compared with non-amended or single treatments (e.g., biochar and compost inputs increased colonization and P acquisition in plants [28]. Rhizobium inoculation combined with soil amendments has been shown to enhance root structure and increase plant nutrient uptake by improving rhizosphere conditions and symbiotic nitrogen fixation efficiency compared with non-inoculated controls [26]. The combined application of AMF, encapsulated *Rhizobium*, and organic amendments enhanced and stimulated microbial functional populations in the rhizosphere. Improved symbiotic efficiency likely facilitated greater nutrient acquisition, resulting in enhanced nutrient uptake[5, 28]. AMF and Rhizobium inoculation enhance auxin-mediated elongation and stem robustness in legumes [8]. These integrated responses contributed to improved plant morphophysiological performance and biomass production [26]. Application of biofertilizers combined with soil organic amendments significantly enhances soil biological activity by stimulating microbial activity, thereby improving nutrient cycling and overall plant performance [26, 34].

## Conclusion

The present study demonstrated that integrating organic amendments with microbial biofertilizers exerts significant synergistic effects on morphophysiological performance, nutrient acquisition, and microbial symbiosis in black soybean (Glycine max) cultivated in Inceptisol. While early growth stages (14 DAP) showed minimal treatment differentiation, pronounced effects emerged from 21 DAP onward, with the strongest responses observed at 28 DAP. Treatments combining Biochar or Vermicompost with dual inoculation (Rhizobium + AMF) consistently enhanced leaf area index, height–diameter ratio, and shoot and root biomass relative to single inoculations or amendment-only controls. Phosphorus uptake and AMF root colonization were markedly improved under combined treatments, with Biochar + dual inoculation achieving the highest colonization (80%) and nitrogen-fixing bacterial population (7.63 × 105 CFU g⁻1 soil). Correlation analysis revealed strong positive associations among growth, nutrient, and microbial parameters.

In contrast, principal component analysis confirmed that coordinated improvements in morpho-physiology, nutrient uptake, and microbial activity accounted for the majority of the treatment variability. Collectively, these findings highlight the potential of integrating Biochar or Vermicompost with dual microbial inoculation as a sustainable strategy to enhance black soybean productivity in nutrient-limited Inceptisol soils. Such combined organic–microbial approaches offer promising avenues for improving crop performance and advancing climate-smart agricultural practices. This study was conducted under controlled experimental conditions using a specific soil type and selected microbial inoculants. Therefore, the findings may not fully represent responses under diverse field environments and agroecological conditions. Future studies should evaluate the long-term field performance of these amendments across different soil types and cropping systems.

## Data Availability

All data supporting the findings of this study are included within the article. No additional datasets were generated or analyzed beyond what is presented in this manuscript.

## Abbreviations

AG: Ground Area
AL: Leaf Area
AMF: Arbuscular Mycorrhizal Fungi
CEC: Cation Exchange Capacity
CFU: Colony-Forming Units
D: Stem Diameter
DAP: Days After Planting
DWᵣ: Root Dry Weight
DWₛ: Shoot Dry Weight
H: Plant Height
HDR: Height-to-Diameter Ratio
KCl: Potassium Chloride
LAI: Leaf Area Index
NFB: Nitrogen-Fixing Bacteria
NL: Number of Leaves
NMNI: Normalized Mean Nodulation Index
P₂O₅: Phosphorus Pentoxide
SPAD: Soil Plant Analysis Development
Weff: Effective Nodule Weight
Wtotal: Total Nodule Weight
Neff: Effective Nodules
Ntotal: Total Nodules
